# Macro- and meso- shear mechanical properties of rock-grout composite structures under different stress level and initial hydration damage

**DOI:** 10.1038/s41598-025-26646-1

**Published:** 2025-11-27

**Authors:** Haoyu Rong, Guichen Li, Jiahui Xu, Dongxu Liang, Feng Lin

**Affiliations:** 1 School of Mines, Key Laboratory of Deep Coal Resource Mining, Ministry of Education of China, China University of Mining and Technology, Xuzhou, China; 2 School of Civil Engineering, Xuzhou University of Technology, Xuzhou, China; 3 State Key Laboratory of Intelligent Deep Metal Mining and Equipment, Northeastern University, Shenyang, China

**Keywords:** Sandstone, Rock-grout composite structure, Hydration, Triaxial shear test, Multiscale, Engineering, Materials science, Solid Earth sciences

## Abstract

This study investigates the shear mechanical behavior of soft rock—grout coupled structures through triaxial shear tests and particle flow simulations on sandstone-resin composite specimens under varying normal stresses and immersion times. Key findings include: (1) Intensified hydration damage compromises shear resistance in both rock and interfaces, amplifies deformation, and degrades bearing capacity. (2) Elevated normal stress constrains microcrack coalescence, enhances bearing capacity. Failure modes of specimens—classified as interfacial shear sliding, mixed shear failure, or shear failure occurs exclusively within rock—depending on stress levels and hydration duration. (3) Prolonged immersion reduces energy thresholds for bond rupture, shifting crack propagation from abrupt surges to gradual increments. Weak zones migrate from interfaces to external rock. (4) Increased normal stress raises energy storage limits, suppresses microcrack coalescence, and strengthens weak zones. This study revealing the critical control of hydration damage and stress confinement on energy thresholds at the rock-grout interface, providing a theoretical basis for long-term stability prediction of anchorage structures.

## Introduction

Soft rock, recognized as the most widely distributed rock type, exhibits inherent geotechnical challenges due to its composition containing unstable components such as clay minerals^1,2^. Characterized by loose structure, poor load-bearing capacity, and pronounced water–rock interactions, this lithology frequently induces critical engineering complications including foundation settlement, slope instability, and significant deformation of underground tunnels^3–6^. These geomechanical behaviors substantially elevate safety hazards and construction complexities in rock-soil engineering projects.

**Fig. 1:**
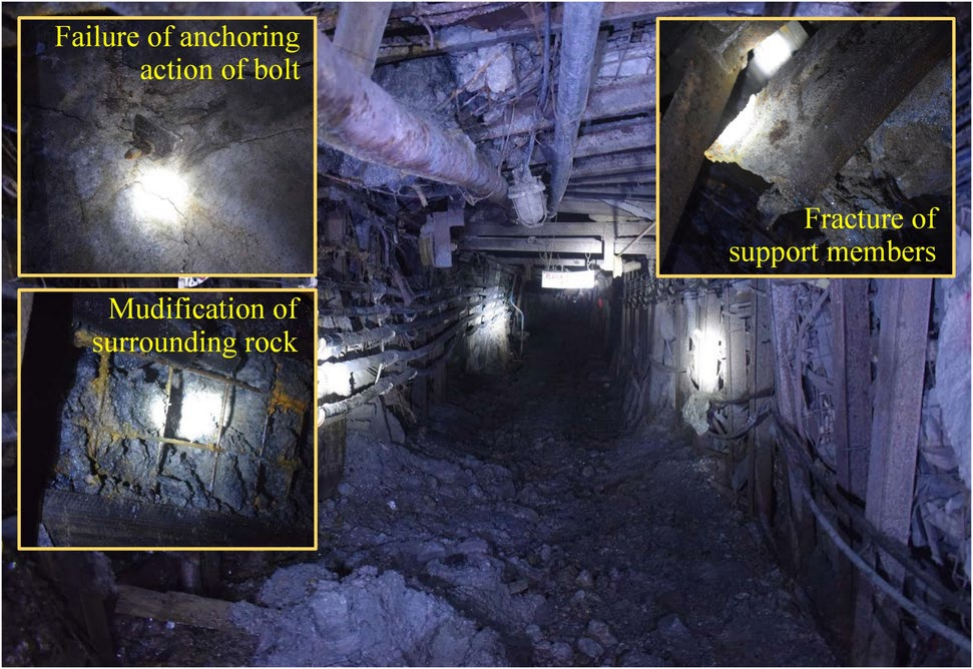
Failure of anchorage structure in soft rock roadway.

Rock bolt support, characterized by cost-effectiveness and high reliability, has been extensively employed as a predominant reinforcement method in underground engineering. This technique has consequently served as the foundation for developing integrated support systems, including shotcrete-mesh-bolt composite support and bolt-truss reinforcement. These advanced synergistic methodologies substantially enhance the stability of underground chambers through optimized load transfer mechanisms and composite structural behavior^7–10^. As shown in Fig. 1, a rock bolt is normally bonded to the surrounding rocks by means of borehole grouting or using anchoring agents, and pre-tightening force is exerted to form active support of the rocks. In a soft rock formation, owing to poor bearing capacity of the surrounding rocks, the anchoring ability of the rocks deteriorate rapidly when exposed to water^11,12^. This weakens the bonding performance of the bolting system. Meanwhile, due to the impact of hydration, a large number of fractures and pores may occur in the surrounding rocks, the integrity of which can be significantly reduced^12–14^. Consequently, the shallow and deep surrounding rocks are hard to form a collaborative bearing structure through rock bolting^15,16^. Therefore, the overall disintegration of the surrounding rocks and the slip of the rock-grout interface are the main forms of rock bolting failure in soft rock cavities^17,18^.

In order to obtain the failure mechanisms of soft rock bolting structures, scholars have conducted a large amount of research and also achieved fruitful results on the dynamic and static mechanical responses and disintegration characteristics of soft rocks, as well as on the supporting mechanism and pull-out performance of rock bolts. Sakai and Nakano^19^ investigated the mechanical behavior of mudstone under wetting–drying cycles, revealing a progressive reduction in particle size with increasing cycle frequency, which directly correlates with strength deterioration through microstructural alteration. Wong et al.^20^ systematically analyzed hydration processes on rock mechanical responses, substantiating that sedimentary rocks exhibit heightened sensitivity to moisture fluctuations compared to igneous and metamorphic counterparts, attributed to their porous fabric and reactive mineral assemblages. Gautam and Shakoor^21^ elucidated disintegration-erosion dynamics of various lithologies under natural climatic exposure, demonstrating that clay-bearing lithologies experience accelerated degradation mechanisms under humidity variations, manifesting as rapid surface exfoliation and mass loss. Zhang et al.^22^ conducted disintegration susceptibility experiments, empirically validating the applicability of Weibull distribution models in quantifying particle fragmentation evolution, thereby establishing probabilistic prediction frameworks for rock disintegration processes. Liang et al.^23^ investigated debonding failure mechanisms in full-length anchorage systems through interfacial mechanics modeling between rock bolts and grouting materials, revealing that when surrounding rock tensile strength falls below that of the grout annulus, structural failure primarily occurs along the grout-rock interface or via rock mass pull-out, driven by stress concentration effects and interfacial stress redistribution. YOKOTA Y^24^ conducted numerical investigations via Discontinuous Deformation Analysis (DDA) to elucidate the parametric sensitivity of rock bolt geometric configurations on interfacial mechanical behavior at bolt-grout interfaces. The study quantitatively established that variations in bolt diameter-to-spacing ratio govern failure mechanisms through interfacial stress transfer efficiency.

However, isolated investigations focusing solely on mechanical responses of rock mass and bolts within anchorage systems fail to capture the complex failure mechanisms inherent in soft rock reinforcement. The rock-grout composite interface emerges as the critical weak link in soft rock anchoring structures, where interfacial load-transfer synergy governed by differential deformation compatibility dictates global stability through dynamic stress redistribution and progressive interface deterioration. Moreover, since the instability of anchorage structures originates from the progressive development of mesoscale damage to macroscopic failure, their mesomechanical properties play a critical role in structural performance. However, constrained by current experimental techniques, conventional macro-mechanical testing approaches face inherent limitations in capturing the damage evolution process at mesoscopic scales, as well as monitoring real-time stress distribution patterns and kinematic deformation behavior across different internal locations of rock specimens during loading processes.

Accordingly, this study investigates the soft rock-grout composite structures through integrated experimental and numerical approaches. The research employed triaxial shear testing under controlled normal stress levels and water immersion durations, combined with particle flow simulations, to investigate the progressive evolution of shear mechanical properties under combined stress states and hydration-driven damage mechanisms. The investigation quantifies failure modes, damage progression characteristics, and internal stress redistribution mechanisms. Ultimately elucidating the macro-mesoscopic failure mechanisms governing soft rock bolting structures instability under coupled stress-hydration interactions.

## Materials and methods

### Specimen preparation

**Fig. 2:**
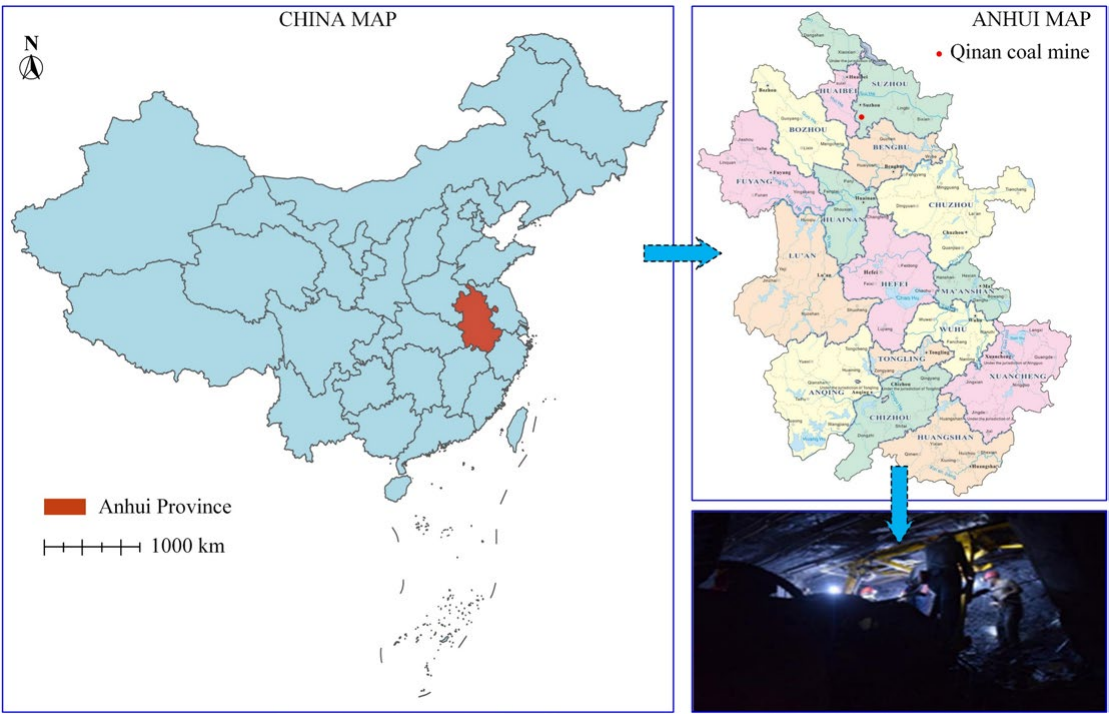
Geographic location of sandstone samples.

The rock samples were collected from Qinan coal mine at Suzhou, Anhui Province, China (as shown in Fig. 2). The lithology was sandstone of the Permian Shanxi Formation, having a fine-grained sand-like structure, a uniaxial compressive strength of 35.25 MPa, an elastic modulus of 2.08 GPa and a Poisson’s ratio of 0.16. The mineral contents in the samples are listed in Table 1.

**Table 1:** Mineral composition of sandstone.

Mineral composition	Quartz	Calcite	K-feldspar	Albite	Montmorillonite	Illite	Chlorite
Content/%	50	12	1	18	9	4	6

**Fig. 3:**
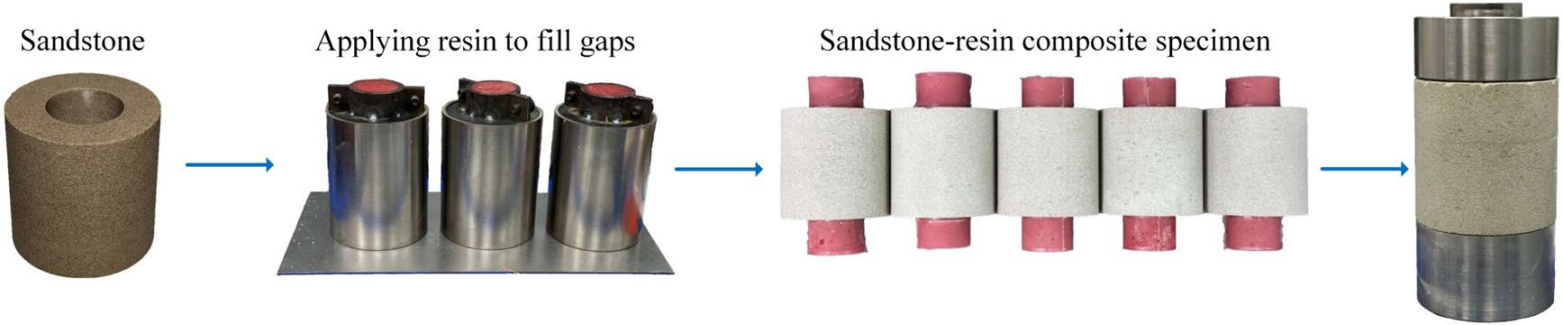
Preparation of sandstone-resin composite specimen.

**Fig. 4:**
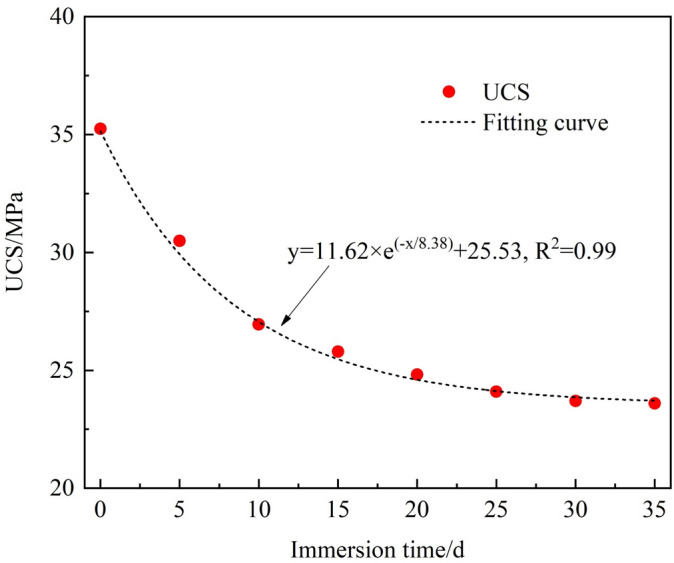
Relationship between UCS of sandstone with immersion time.

**Fig. 5:**
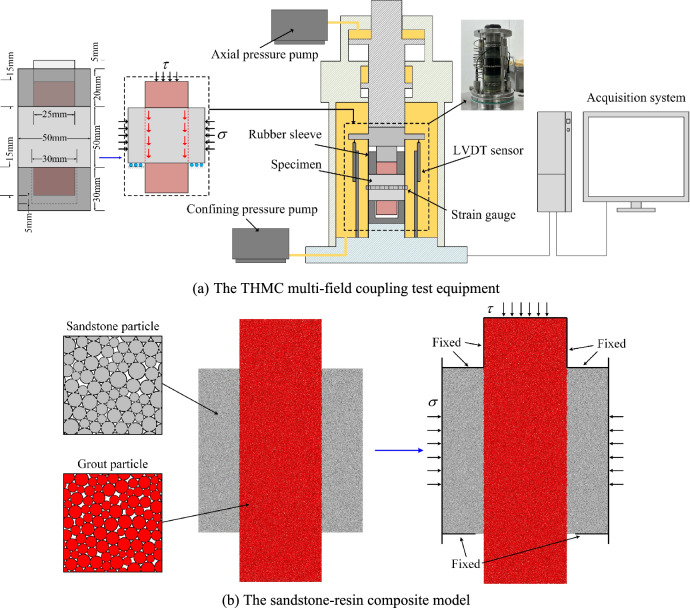
Test system.

The specimens for the experiment were cut from the same uniform rock block. Standard cylindrical specimens with a size of *Φ*50 mm × 100 mm were processed. Annular specimens with an outer diameter of 50 mm, an inner diameter of 25 mm and a height of 50 mm were also prepared. The errors of specimen heights and diameters were both within ± 0.5 mm, and the parallelism error of the rock specimen surfaces was within ± 0.02 mm. Acoustic wave velocity tests were conducted to eliminate the specimens with obvious defects and high dispersion of wave velocity. The sandstone specimens were baked in a drying oven. Since the sandstone contained crystal water (montmorillonite), the temperature was set at 65 ºC and the specimens were baked for 12 h^25^. The dried sandstone specimen was placed in a mold, and then grout materials were poured into the mold and waited for solidification. Epoxy resin with similar mechanical properties to mine-used grout was selected as the grout. Its curing time was 0.5 h and the average uniaxial compressive strength after solidification was 47.68 MPa, satisfying the test requirements. Specimen size and preparation process are shown in Fig. 3. After solidification of the resin, the sandstone-resin composite specimens were packaged into a container and vacuumized at the pressure of 0.1 MPa for 12 h. Thereafter the specimens were soaked in the aqueous solution and strictly sealed. To simulate the real immersion environment of rock-grout structures, aqueous solution was collected from Qinan coal mine for subsequent immersion of specimens. The major cations in the groundwater were $Na^{ + }$, $Ca^{2 + }$ and $Mg^{2 + }$, the main anions were $SO_{4}^{2 - }$, $Cl^{ - }$ and $HCO_{3}^{ - }$, and the *pH* value was within 8.0 ± 0.5. The dimensions of the numerical model were maintained consistent with those of the experimental specimens, with particle diameters randomly generated within the range of 0.1–0.2 mm. Fig. 4 and 5

The THMC (thermo-hydro-mechanical-chemical) multi-field coupling test equipment was employed to perform the triaxial shear tests. During the shearing process, different confining pressures were exerted on the side surface of the sandstone specimen, axial loads were applied on the top of the grout, and the confining pressure along the sandstone annulus was equivalent to the normal stress in a simple shear test. Hyett et al.^26^ concluded that the normal pressure of the borehole wall resulted by engineering disturbance and stress redistribution after rock bolt installation does not exceed 10 MPa, so the normal stress (*σ*) in the current study was set to be 2, 4, 6, 8 and 10 MPa, respectively. In the test, *σ* was firstly applied to the designed value at a rate of 1 MPa/min by means of stress control, and then shear displacement was applied at a rate of 0.05 mm/min by means of displacement control until the specimen failed.

### Parameter calibration

**Fig. 6:**
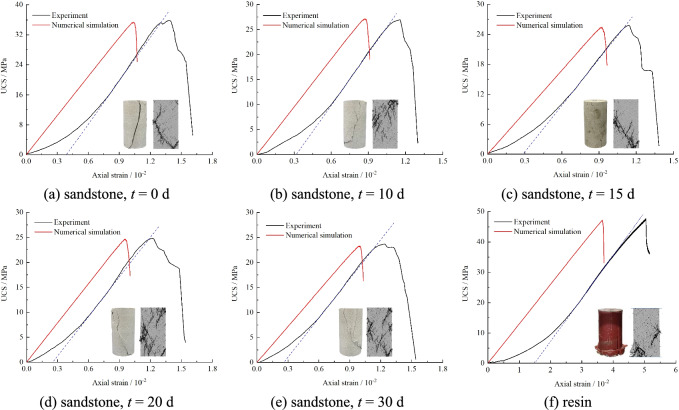
Stress–strain curve obtained from simulation and test.

Parallel-bond model is a commonly used as the contact model for simulating rock-like materials. The failure of the parallel-bond model will lead to the rapid reduction of macroscopic stiffness of the model, which is consistent with the failure mechanism of rock material^27^. Therefore, the parallel-bond model is used in the simulation of this paper. The initial model was generated and saved to ensure that all subsequent steps were performed under the same initial conditions. Following model establishment, wall elements in PFC were employed to apply loads, with the simulated loading path rigorously aligned with experimental procedures. Since laboratory tests employ quasi-static loading, a loading rate of 0.1 m/s is adopted in the PFC simulations. This rate falls within the range of 0.016–0.2 m/s, which satisfies quasi-static conditions and exhibits no significant effects on simulation results^28^. As shown in Table 2, the mesoscopic parameters of the model were determined through trial-and-error calibration based on uniaxial compression test results of resin and sandstone specimens with varying water immersion durations. As can be seen from the figure, the results obtained by PFC simulation are consistent with the experimental results, and the selected parameters can better capture the mechanical properties of the resin and mudstone, as shown in Fig. 6.

**Table 2:** Meso-parameters of numerical model.

Material	Minimum radius of the particle *R*_*min*_/mm	Ratio of maximum to minimum of radius *R*_*max*_*/R*_*min*_	Young’s modulus of the particle *E*_*c*_/GPa	Ratio of normal to shear stiffness of the particle k*	Friction coefficient *μ*	Young’s modulus of the parallel bond $\overline{E}_{c}$/GPa	Ratio of normal to shear stiffness of the parallel bond $\overline{k}^{*}$	Tensile strength of parallel $\overline{\sigma }_{c}$/MPa	Shear strength of parallel $\overline{c}$/MPa	*t*/d
Sandstone	0.1	2	1.5	1.0	0.58	1.5	1.0	12.0	15	0
			1.3	1.1	0.55	1.3	1.1	8.0	12	10
			1.2	1.3	0.52	1.2	1.3	7.5	12	15
			1.1	1.5	0.51	1.1	1.5	6.0	12	20
			1.0	1.6	0.51	1.0	1.6	6.0	11	30
Resin	0.1	2	0.6	1.1	0.58	0.6	1.1	15.0	20	–-

## Test results

### Deformation characteristics of specimens

**Fig. 7:**
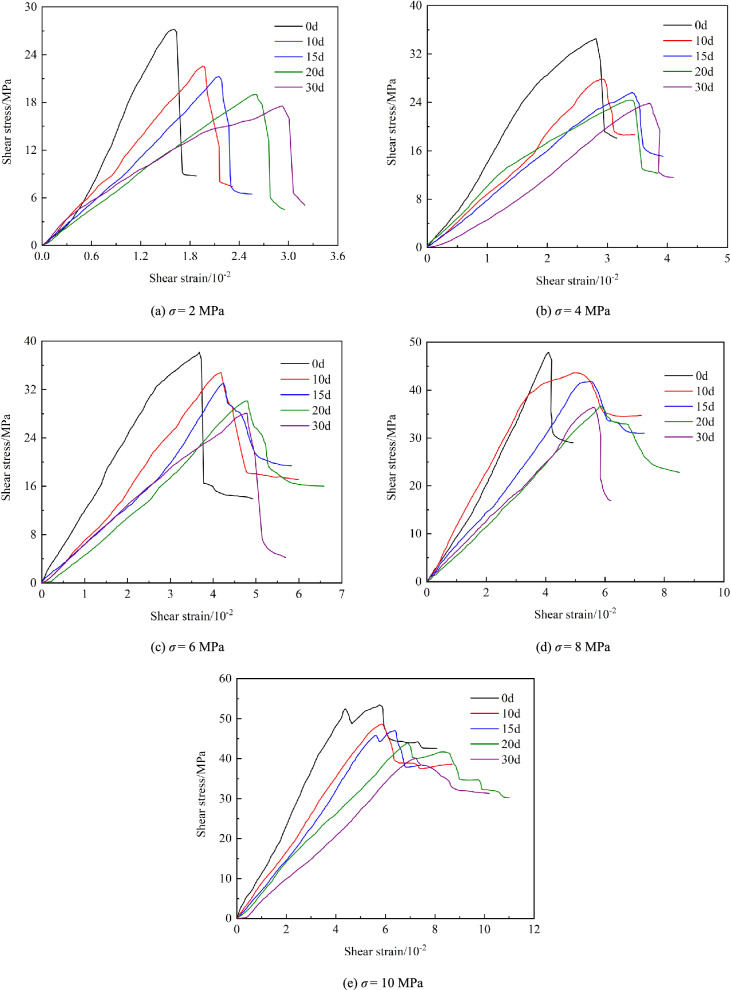
Shear stress − displacement curves of specimens.

Fig. 7 presents the relationship between the shear stress (*τ*) and the shear displacement (*u*) at varying immersion times and *σ*. It can be seen that the shear stress-displacement curves have the same variation trend. Before peak stress, *τ* demonstrates linear growth with *u*. Nonlinear characteristics emerge near peak stress, accompanied by micro-fracture generation and propagation within specimens under shear stress. Post-peak behavior shows rapid stress reduction as undeveloped sections undergo accelerated failure along shear planes, resulting in loss of cooperative load-bearing capacity between rock and grouting material. However, residual shear resistance persists due to frictional effects at shear interfaces influenced by normal stress.

**Fig. 8:**
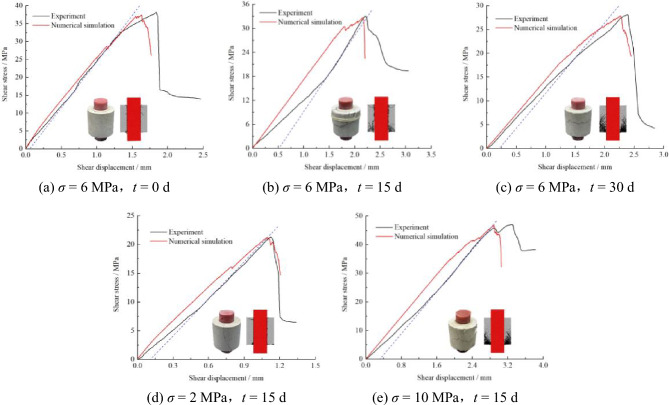
Stress–strain curve obtained from simulation and test.

To validate simulation accuracy, experimental and numerical curves under *σ* = 6 MPa with *t* = 0, 15, 30 d, and *t* = 15 d with *σ* = 2, 6, 10 MPa are compared in Fig. 8. The PFC loading mechanism induces model self-equilibrium through compaction during initialization, yielding uniform particle distribution that causes inherent simulation-experiment deformation discrepancies^28,29^. Notably, simulated stress-deformation relationships and failure characteristics closely match experimental results. The numerical model effectively replicates key mechanical responses despite minor deviations in deformation magnitudes.

### Failure mode

**Fig. 9:**
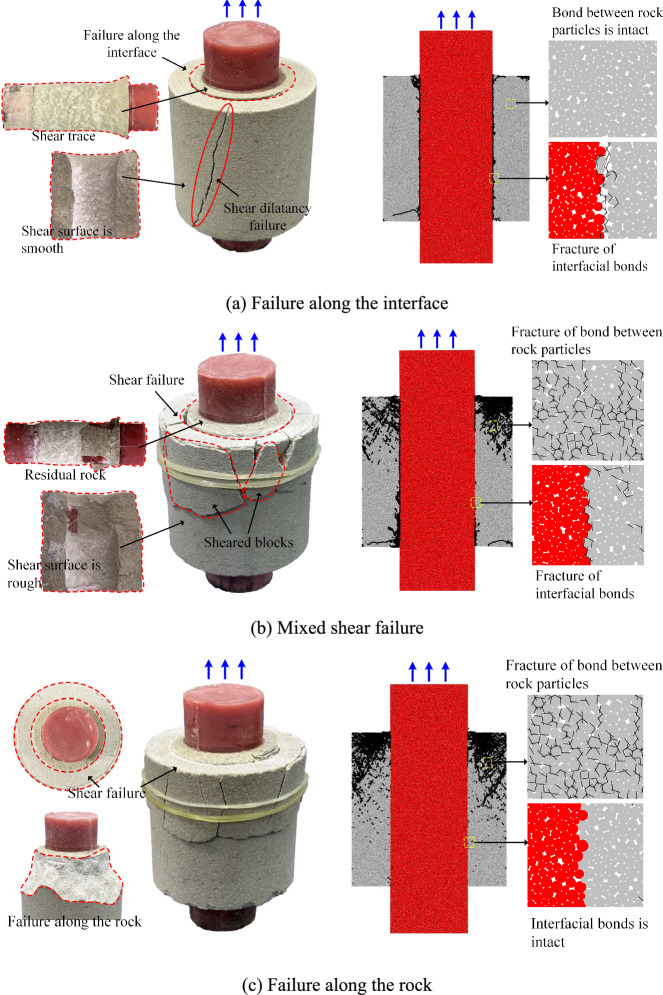
Typical shear failure modes of the specimens.

Fig. 9 illustrates typical failure modes of specimens, with blue arrows indicating shear loading directions. Black lines in PFC images represent microcracks formed by bond breakages. As sandstone strength and rock-grout interface bonding properties vary with *σ* and immersion duration, weak zones within specimens exhibit corresponding modifications. Consequently, distinct shear failure patterns emerge under different testing conditions.

Based on the experimental observations, three primary failure modes are identified in the specimens. Failure Mode I: Shear failure occurs along the grout-rock interface, accompanied by radial fracturing in rock material. Micromechanical simulations reveal bond fractures predominantly at the grout-rock contact and between rock particles near the interface. Microcracks concentrate at the bonding interface, while sandstone interior maintains intact particle bonds. Consequently, a thin rock powder layer adheres to the grout surface, with smooth shear planes and distinct slickensides observed. The grout-rock interface constitutes the weak zone in this failure mechanism.

Failure Mode II: The fracture surface comprises both grout-rock interface and rock, exhibiting hybrid shear failure. Shear-induced bond fractures occur between rock-grout particles and within rock particles. Notably, intensive bond fractures develop among rock particles at the shear-loaded end. This results in residual rock fragments adhering to the grout surface and blocky fractures forming at the sandstone top under shear. The weak zone combines interface and rock matrix components. Failure Mode III: Shear failure occurs exclusively within rock while preserving grout-rock interface integrity. Rock-grout bonds remain intact, whereas extensive bond fractures occur between rock particles at the loaded end. Microcracks predominantly propagate within sandstone, creating shear surfaces with significant morphological and roughness variations. The weak zone localizes in rock material adjacent to the grout body.

Figu. 10 illustrates the distribution of specimen failure modes. At *σ* ≤ 4 MPa, all specimens exhibited Mode I failure, indicating higher shear strength in rock than at the grout-rock interface. With increasing immersion duration, degradation of bonding capacity and frictional resistance between rock and grout progressively reduced specimen shear strength. Within the range of 6 MPa ≤ *σ* ≤ 8 MPa, failure modes transitioned sequentially from Mode I to Mode III. At *σ* = 10 MPa, all specimens failed exclusively through Mode III. Notably, under constant *σ*, the load-bearing capacity of rock degraded more significantly than that of the grout-rock interface with prolonged immersion. To elucidate failure mechanisms, sandstone fracture surfaces under varying immersion durations were analyzed using ZEISS Sigma 300 scanning electron microscopy (SEM), as shown in Fig. 11.

**Fig. 10:**
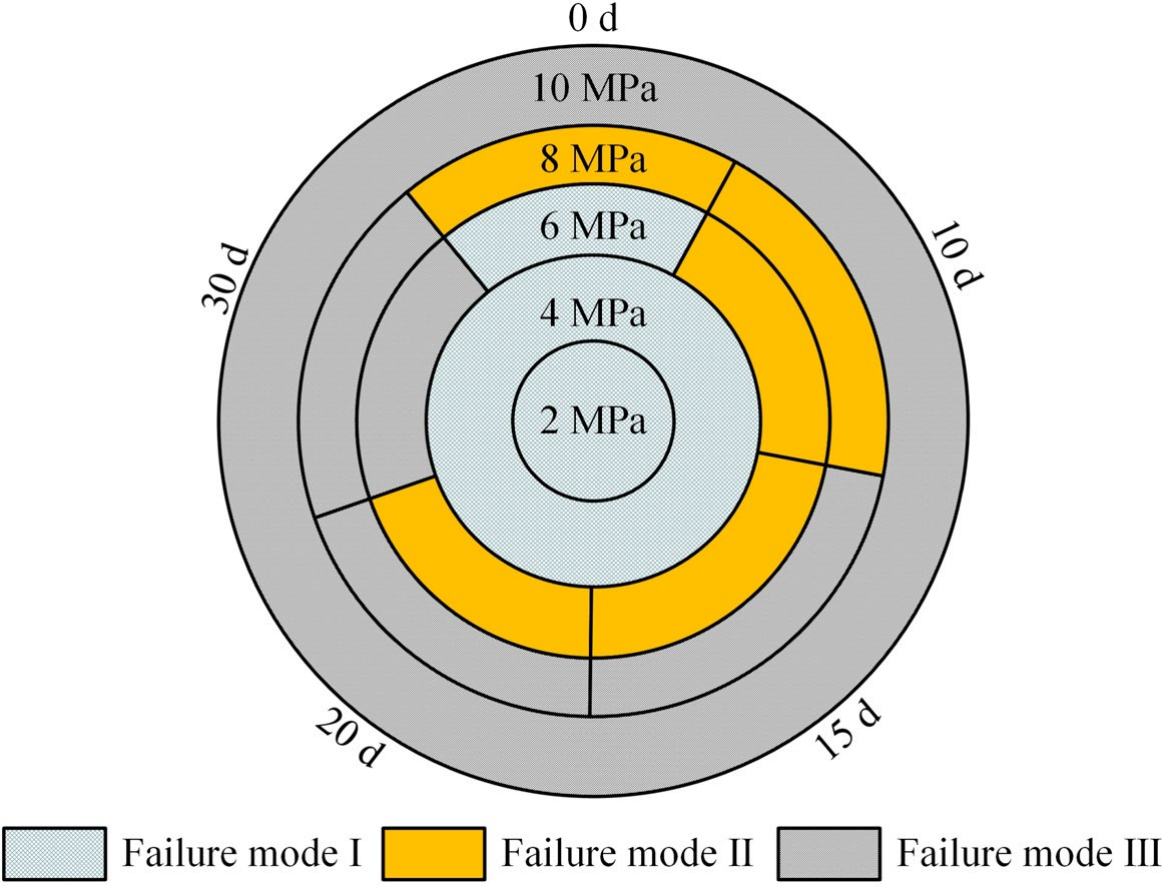
Distribution of failure modes.

**Fig. 11:**
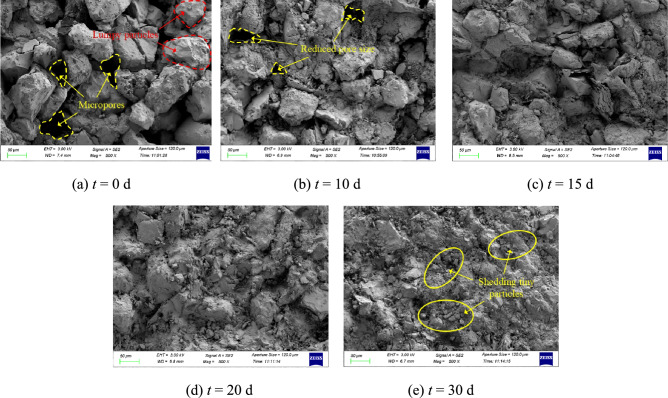
The SEM images for sandstones with various immersion times.

As shown in Fig. 11, under dry conditions, sandstone surfaces exhibit blocky mineral particles with observable micro-pores. XRD results confirm that inter-detrital cementation dominates the sandstone, supplemented by clay-clay and detrital-clay particle bonding. These micro-pores between detrital particles facilitate grout penetration, enabling effective bonding with surface minerals to form load-sharing structures. Prolonged immersion degrades inter-detrital cementation and induces clay particle gelation. Hydration triggers ion exchange in potassium feldspar and albite, generating kaolinite with enhanced dispersibility and softening. Montmorillonite, illite, and chlorite undergo gelation and swelling, progressively clogging surface pores. Post 30-day immersion, extensive detached fine mineral debris is observed on sandstone surfaces. Complete pore occlusion eliminates observable micro-porosity. Concurrent clay mineral gelation induces surface particle disintegration, impeding effective grout-rock bonding and triggering mechanical properties of interface degradation. Consequently, under constant *σ*, prolonged immersion exacerbates initial sandstone damage, accelerating rock strength deterioration. Increased *σ* enhances frictional resistance at the grout-rock interface while compacting incompatibility spaces and shear-induced microcracks, thereby strengthening the interface. This shifts the weak zone from the interface to the rock. Notably, the increase in *σ* enhances the strength of the interface more significantly than it does the rock strength.

### Fracture development characteristics

This section examines simulation results under *σ* = 6 MPa with immersion durations *t* = 0, 15, and 30 d, as well as *t* = 15 d with *σ* = 2, 6, and 10 MPa. Microcrack propagation trends across distinct failure modes were analyzed to characterize fracture development.

**Fig. 12:**
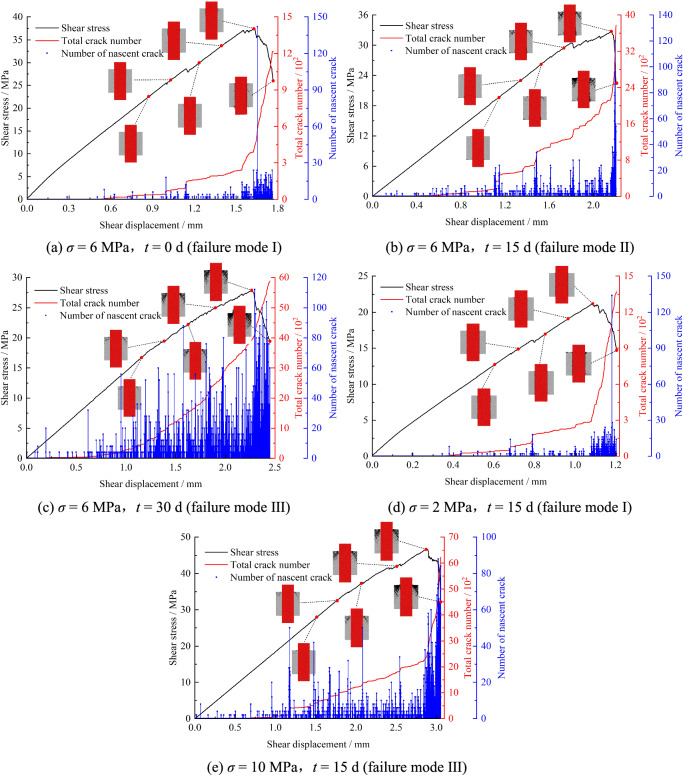
Relationship between shear stress, the total crack number, and the number of nascent crack.

Fig. 12 presents the relationship between shear stress, total microcrack count, and newly generated cracks. Six characteristic stages (*t*_*1*_ – *t*_*6*_) were analyzed, corresponding to 60%, 70%, 80%, and 90% of pre-peak stress, peak stress, and 70% of post-peak stress, respectively. As shown, the growth rate of total microcracks accelerates with rising shear stress across all conditions, with new crack generation peaking near peak shear stress. For *σ* = 6 MPa and *t* = 0 d, microcrack development remains gradual before *t*_*4*_, showing minimal fluctuation in new cracks.

**Fig. 13:**
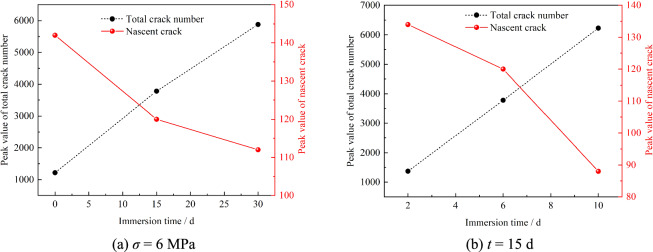
Peak value of total crack number and nascent crack.

Rapid microcrack propagation and a new crack peak occur during *t*_*5*_ – *t*_*6*_, indicating coalescence-driven macroscopic failure and subsequent shear stress decline. Prolonged immersion accelerates total microcrack growth rates. At *σ* = 6 MPa and *t* = 15 d, total microcracks exhibit accelerated growth from stage *t*_*3*_. Under *σ* = 6 MPa and *t* = 30 d, parabolic growth of total microcracks and pronounced fluctuations in new crack counts are observed. Fig. 13(a) shows total microcrack peaks of 1216, 3779, and 5879 for *σ* = 6 MPa at *t* = 0, 15, and 30 d, respectively, with new crack peaks of 142, 120, and 112. Extended immersion increases total microcrack accumulation while amplifying fluctuations of newly generated curve, albeit with reduced amplitude. Similar trends emerge under varying normal stress. Fig. 13(b) displays total microcrack peaks of 1372, 3779, and 6228 for *t* = 15 d at *σ* = 2, 6, and 10 MPa, respectively, alongside new crack peaks of 134, 120, and 88. Higher normal stress enhances total microcrack development but suppresses new crack generation.

Prolonged immersion elevates initial hydration-induced damage in specimens, triggering bonding capacity degradation between particles. This process lowers the energy threshold for bond fracture, facilitating microcrack propagation and interconnection. Consequently, fracture evolution transitions from an abrupt surge to a gradual progression. In contrast, increased normal stress suppresses microcrack coalescence, necessitating higher microcrack density to initiate macroscopic failure.

## Shear stress evolution of the model

**Fig. 14:**
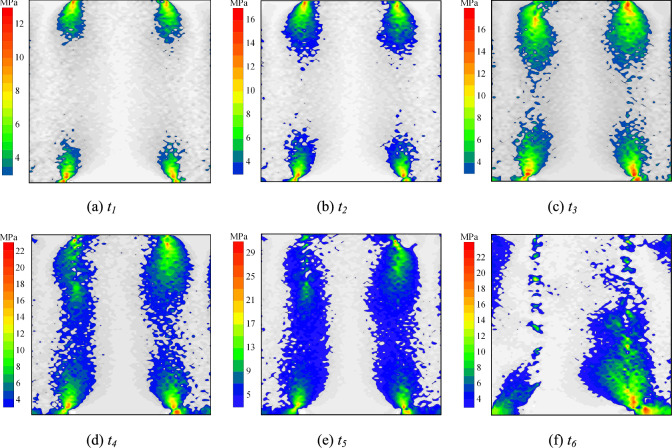
Particle shear stress distribution at condition of *σ* = 6 MPa and *t* = 0 d (failure mode Ⅰ).

**Fig. 15:**
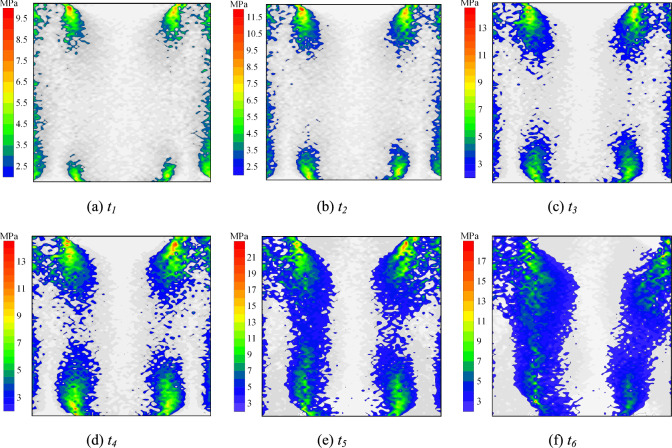
Particle shear stress distribution at condition of *σ* = 6 MPa and *t* = 15 d (failure mode Ⅱ).

**Fig. 16:**
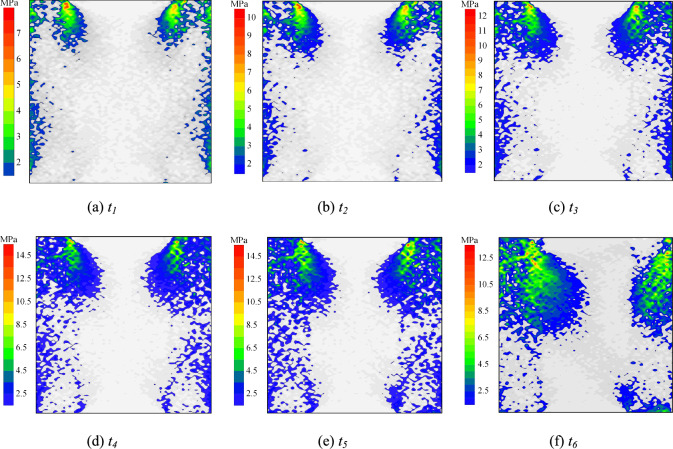
Particle shear stress distribution at condition of *σ* = 6 MPa and *t* = 15 d (failure mode Ⅲ).

**Fig. 17:**
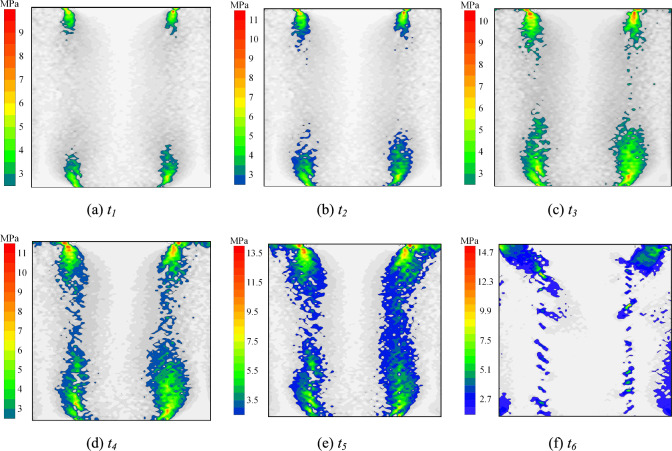
Particle shear stress distribution at condition of *σ* = 2 MPa and *t* = 15 d (failure mode Ⅰ).

**Fig. 18:**
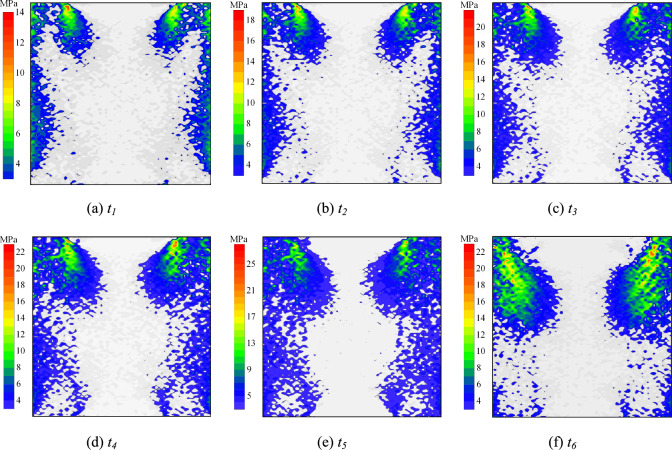
Particle shear stress distribution at condition of *σ* = 10 MPa and *t* = 15 d (failure mode Ⅲ).

Experimental results demonstrate three primary failure modes in specimens. This section examines the meso-scale damage evolution mechanisms of rock-grout composite structures by analyzing shear stress distribution patterns among internal particles under distinct failure modes. Fig. 14–18 present the shear stress distributions of specimen particles, excluding the exposed grout sections. The shear direction is consistent with that shown in Fig. 5(b).

Fig. 14 displays the shear stress distribution of specimen particles under *σ* = 6 MPa and *t* = 0 d (Failure Mode I). For clarity, the exposed grout segment is excluded, and areas with shear stress below 10% of the peak particle shear stress are desaturated to grayscale. Stages *t*_*1*_ – *t*_*6*_ correspond to 60%, 70%, 80%, and 90% of pre-peak stress, peak stress, and 70% of post-peak stress on the stress–strain curve, respectively.

During the pre-peak stage, shear stress concentration zones initially form at both ends of the interface, progressively propagating toward the specimen center with increasing shear displacement. Concurrently, peak particle shear stress intensifies, ultimately developing a stress concentration belt centered on the interface that encompasses surrounding rock and grouting material. The interface serves as the critical load-bearing zone, synergistically interacting with adjacent materials to establish a cooperative load-transfer mechanism. In the post-peak stage, interfacial bond fractures at the interface progressively diminish its load-transfer coordination. This leads to continuous shrinkage of particle shear stress concentration zones, accompanied by stress migration toward the rock and grouting material domains. Concurrently, peak particle shear stresses exhibit progressive attenuation, ultimately culminating in failure of the specimen.

Fig. 15 illustrates the particle shear stress distribution (Failure Mode II) under *σ* = 6 MPa and *t* = 15 d. The stress evolution follows patterns consistent with previous observations, yet manifests as an inclined shear stress concentration band incorporating both the interface and the top of the rock. The critical load-bearing system transitions to a composite structure comprising the interface and peripheral rock. In the post-peak stage, interfacial debonding and rock bond fractures induce progressive deterioration of this bearing system. This degradation mechanism drives systematic attenuation of particle shear stresses, ultimately resulting in global structural collapse.

Fig. 16 displays the particle shear stress distribution (Failure Mode Ⅲ) under *σ* = 6 MPa and *t* = 30 d. The shear stress concentration zones predominantly localize within bilateral rock segments at the specimen’s upper region, with the critical load-bearing system transitioning to the rock beyond the grouting material. Under constant normal stress, progressive immersion duration induces attenuation of peak particle shear stresses at same time nodes. Concurrently, weak zones in the rock-grout coupled structure migrate from the interface to peripheral rock, accompanied by continuous degradation of their load-bearing capacity. Notably, shear strength deterioration rates in the rock exceed those observed at the interface.

Combined with Fig. 11, prolonged water immersion leads to continuous reduction in the shear strength of sandstone due to the swelling and slaking of clay minerals. Simultaneously, the slaked clay minerals inhibit full bonding between the grout and rock particles. Under constant normal stress, the particle shear stress peak at any given time decreases with extended immersion, resulting in a progressive decline in the load-bearing capacity of weaker zones in the specimen. However, due to the residual bonding effect of the grout on rock particles, the strength degradation at the interface occurs at a slower rate compared to the rock. Consequently, the weaker zones gradually shift outward from the interface.

Fig. 17 and 18 present shear stress distribution of particles under *t* = 15 d with *σ* = 2 MPa and 10 MPa, respectively. With constant immersion duration, increasing normal stress enhances particle shear stress peaks at same time points. At *t*_*5*_, the shear stress peaks under *σ* = 6 MPa and 10 MPa show 55.56% and 85.19% increments compared to *σ* = 2 MPa. The load-bearing limit of weak zones expands with elevated normal stress. Under *σ* = 2 MPa, the shear stress concentration zone exhibits accelerated decay in spatial extent during the post-peak stage. As normal stress increases, the load-bearing capacity enhancement of interfaces outpaces that of the rock. Consequently, weak zones progressively migrate from interfaces to peripheral rock.

It is observed that increased normal stress densifies the specimen. This enhances friction among rock particles and between rock and grout particles. Consequently, the constraint on interparticle movement and deformation is improved. The particle shear stress peak thus rises with increasing normal stress. The load-bearing capacity of weaker zones in the specimen is thereby enhanced. Under *σ* = 2 MPa, the shear stress concentration zone decays more rapidly in the post-peak stage. Meanwhile, increased external shear load intensifies the shear dilation effect in the grout. This further augments friction and mechanical interlocking at the interface. Therefore, the increase in load-bearing capacity at the interface surpasses that of the rock. The weaker zones consequently shift gradually from the interface toward the rock.

## Discussion

**Fig. 19:**
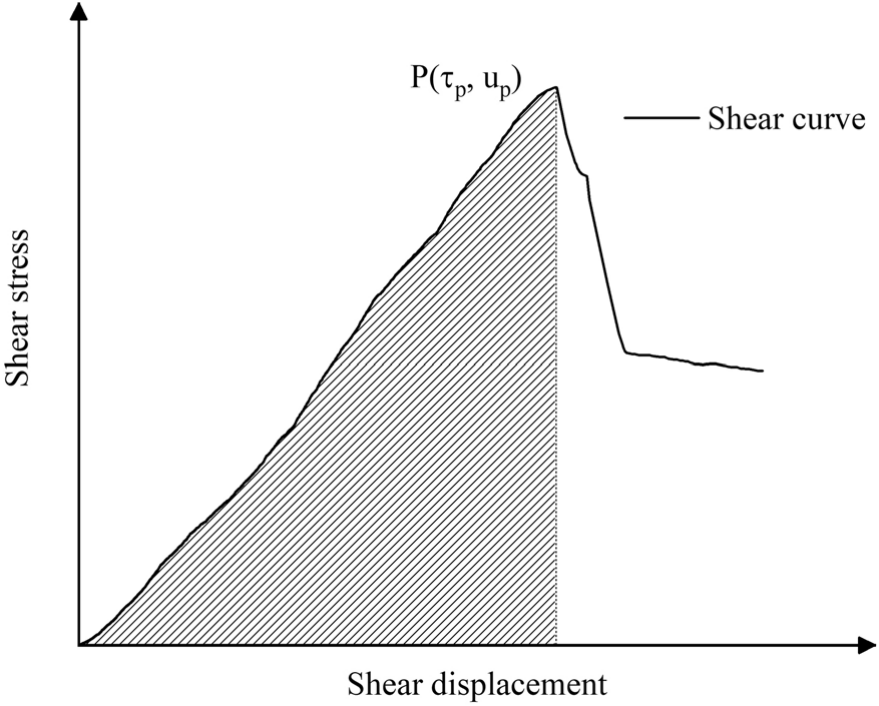
Specimen shear energy absorption schematic.

The failure of anchored structures results from the cumulative effects of energy accumulation and transformation, primarily involving strain energy and dissipated energy. Shear energy quantifies internal energy variations during shear deformation of rock-grout composite specimens under shear forces^11,30^. As shown in Fig. 19, Point P marks the peak shear stress in the curve. The shaded area represents the actual pre-peak energy absorption value of the specimen, denoted as *E*_*p*_.

The bearing capacity and damage evolution of rock-anchorage coupled structures exhibit significant variations with immersion duration and normal stress. This demonstrates that their energy storage limits and energy evolution are governed by these two factors. The bearing capacity and damage evolution of rock-anchorage coupled structures exhibit significant variations with immersion duration and normal stress. This demonstrates that their energy storage limits and crack-induced dissipated energy are governed by these two factors. To characterize energy evolution patterns under different conditions, an energy storage coefficient *D*_*p*_ = *E*_*p*_/u_p_ is introduced. Higher *D*_*p*_ values indicate greater energy storage capacity, with strain energy constituting a larger proportion of shear energy-converted energy. Conversely, lower *D*_*p*_ reflects increased dissipated energy dominance in the shear-induced energy conversion.

**Fig. 20:**
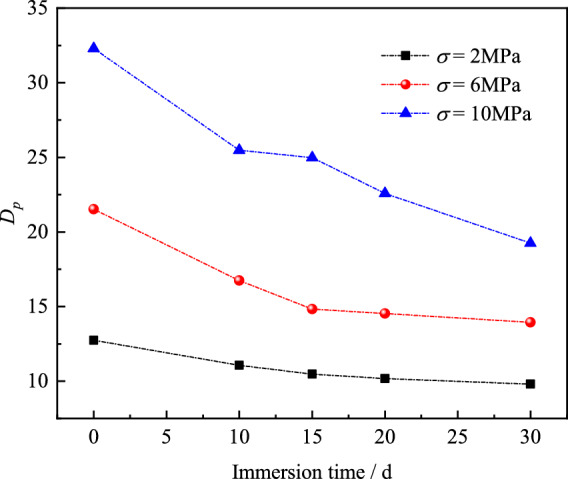
*D*_*p*_ of specimens under different condition.

This section analyzes *D*_*p*_ variations under *σ* = 2, 6, and 10 MPa using experimental data (Fig. 20). Results show *D*_*p*_ increases with normal stress but decreases with prolonged immersion. Extended immersion promotes damage propagation, elevating dissipated energy proportion in shear energy conversion and reducing energy storage capacity. Conversely, higher normal stress suppresses crack coalescence, decreasing dissipated energy proportion. This enhances strain energy conversion efficiency, progressively improving energy storage limits of specimens.

This study investigates the mesoscopic energy evolution in particle flow code (PFC) simulations under *σ* = 2, 6, and 10 MPa. The dissipated energy comprises particle kinetic energy (*E*_*c*_) and sliding friction energy (*E*_*μ*_). The strain energy contains the parallel bond strain energy (*E*_*k*_). Energy transformation mechanisms were systematically analyzed under varying immersion times and normal stresses.

**Fig. 21:**
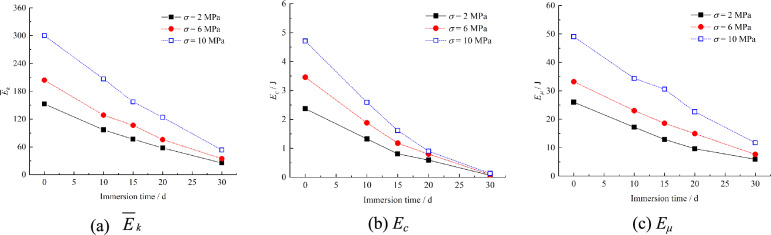
Energy evolution of specimens under different condition.

Figure 21 presents energy evolution curves under varying normal stresses and immersion times. All curves exhibit consistent trends: both strain energy and dissipated energy increase with higher normal stress but decrease with prolonged immersion. Enhanced normal stress elevates the specimen’s energy storage capacity, requiring greater energy input to induce bond fracture and particle movement, thereby improving mechanical strength. Conversely, intensified initial hydration damage reduces energy storage capacity, lowers energy thresholds for bond failure and particle motion, and promotes microcrack initiation and coalescence, ultimately weakening the specimen.

## Conclusion

This study investigates the macro- and meso- mechanical properties of soft rock—grout coupled structures through triaxial shear tests and numerical simulations under varying normal stresses and immersion times. Key findings are summarized as follows:

(1) Intensified initial hydration damage weakens interfacial bonding efficacy, reduces frictional resistance, and induces rock softening. Shear strength of the specimen decreases with prolonged immersion. It increases the micro-fracture density required for macroscopic failure, thereby enhancing both load-bearing capacity and pre-failure deformation.

(2) Shear failure modes of the coupled structure are categorized into three types: shear failure along the interface, mixed shear failure, and failure within the rock. These modes are governed by the combined effects of normal stress and immersion duration.

(3) Prolonged immersion degrades bonding capacity of particles. This facilitates microcrack initiation and coalescence, shifting crack propagation from abrupt surges to gradual increments. Conversely, increased normal stress inhibits microcrack coalescence, raising the critical microcrack density required for macro-failure.

(4) Enhanced initial hydration damage reduces peak shear stress in particles. It shifts weak zones from the interface to adjacent rock while diminishing their bearing capacity. Higher normal stress amplifies load-bearing improvement at interface over rock, progressively transferring weak zones toward external rock regions. Normal stress enhances bearing capacity more effectively at interface than in rock.

(5) Elevated normal stress enhances the specimen’s energy storage limit and elevates energy demands for bond fracture. This increases external energy input required for failure. Conversely, intensified initial hydration damage reduces energy storage capacity, lowers energy thresholds for bond rupture and particle motion, and promotes microcrack nucleation-coalescence.

(6) Since the rock specimens in this study are soft rocks with a clay mineral content of no more than 25%, the applicability of our findings to mudstone (clay mineral content > 25%) and rocks free of clay minerals (hard rocks and some soft rocks) warrants further investigation.

## Data Availability

Data will be made available on request.
